# Pregnancy complications and caesarean section: a latent class analysis

**DOI:** 10.7189/jogh.16.04099

**Published:** 2026-04-03

**Authors:** Tingting Jiang, Ge Yu, Changgui Kou, Wenjun Li, Yihang Liu, Yujiao Meng, Lixin Wan, Liyan Yi, Wei Bai

**Affiliations:** 1 Department of Health, Jilin Women and Children Health Hospital, Changchun, China; 2 Department of Social Medicine and Health Management, School of Public Health, Jilin University, Changchun, China; 3 Department of Epidemiology and Biostatistics, School of Public Health, Jilin University, Changchun, China

## Abstract

**Background:**

The incidence and overuse of caesarean section, an adverse pregnancy outcome closely associated with pregnancy complications, is markedly high globally. However, previous research has predominantly examined individual complications in isolation, leaving a need for a comprehensive evaluation of multimorbidity patterns. We aimed to explore the associations between caesarean sections and adverse pregnancy outcomes in a Chinese population.

**Methods:**

We retrieved data from the National Maternal Near Miss Surveillance System in Jilin Province in China from 2021 to 2023. We summarised them using descriptive statistics and used the Rao-Scott χ^2^ test to compare the differences between spontaneous labour and caesarean section. Then, we used latent class analysis (LCA) to cluster pregnancy complications and logistic regression to examine their association with modes of delivery.

**Results:**

We included 85 446 pregnant women, of whom 53 916 (63.1%) had undergone caesarean sections and 31 530 (36.9%) had experience spontaneous labour. There were significant differences in terms of pregnancy complications between pregnant women who underwent spontaneous labour and those who had caesarean sections. We then clustered pregnancy complication symptoms into six classes using LCA and fitted three models. After adjusting for potential confounders, the incidence of caesarean sections was significantly higher in pregnant women with diabetes and hypothyroidism (odds ratio (OR)  =  1.177; 95% confidence interval (CI)  =  1.105–1.253), hyperthyroidism and kidney disease (OR = 2.078; 95% CI = 1.391–3.106), with hypertension and hypothyroidism (OR = 3.613; 95% CI = 3.217–4.058), and hypertension, diabetes, and anaemia (OR = 3.365; 95% CI = 2.997–3.779) when compared to pregnant women with a lower incidence of pregnancy complications.

**Conclusions:**

Caesarean sections occur frequently among pregnant women in China and are significantly associated with specific pregnancy complication clusters, particularly those involving hypertension, diabetes, anaemia, and thyroid dysfunction. These findings suggest that women with multimorbidity profiles should receive enhanced antenatal surveillance and individualised delivery planning to optimise maternal outcomes.

Pregnancy complications, such as hypertension, diabetes, and anaemia, occur frequently worldwide and can result in maternal and neonatal mortality and morbidity [1–3]. They often vary across contexts and depend on factors such as demographics and prior experiences of pregnancy [3,4]. They are also closely associated with caesarean sections which, despite being a life-saving intervention, have not shown to lower mortality rates in cases where population-level caesarean section rates exceed 16% [5,6]. In fact, some studies have indicated that caesarean section had short- and long-term adverse effects both on women and their infant [7]. This paradox is particularly acute in Chinese population, where the national caesarean rate far surpasses the WHO-recommended threshold of 15%, with an incidence rate of over 30% [8,9]. However, robust evidence characterising the relationship between specific complication profiles and delivery mode in this population remains scarce.

A fundamental challenge in addressing this overuse is that pregnancy complications rarely occur in isolation [10]. Multiple conditions often co-exist, sharing risk factors or pathophysiological pathways [11]. However, conventional analyses typically examine individual complications separately, potentially obscuring the burden of multimorbidity on clinical decision-making. Latent class analysis (LCA) models offer a methodologically rigorous approach to this problem, as they operate on the premise that the observed distribution of variables results from a finite latent mixture of underlying distributions [12] and are regarded as more statistically robust than usual cluster analyses [13]. Specifically, the approach could be employed to identify homogeneous subgroups within the heterogeneous population of pregnant women with comorbidities. This would allow for the characterisation of distinct multimorbidity patterns and their differential associations with caesarean section utilisation.

Despite the recognised association between caesarean section and pregnancy complications, several gaps remain in our understanding of these conditions in China, where the overall caesarean section rate exceeds the WHO-recommended thresholds [14]. Nevertheless, robust evidence from large-scale Chinese cohorts characterising these associations remains scarce. Secondly, research has predominantly concentrated on medically indicated caesarean sections [8], thereby resulting in an insufficient quantification of the risks associated with non-medically indicated procedures. Thirdly, most prior studies have examined individual adverse outcomes in isolation. With these gaps in mind, we sought to ascertain the prevalence of pregnancy complications in the population of Chinese pregnant women and to examine the associations between various pregnancy complications and the incidence of caesarean sections.

## METHODS

### Data source and participants

We retrieved our study data from the National Maternal Near Miss Surveillance System established by the National Health Commission in China in 2010 to capture annual data from municipal, provincial, and national hospitals onto an online system [15]. Here we used data from 13 hospitals in Jilin Province, spanning the period from 1 January 2021 to 31 December 2023. Our study adheres to the Journal of Global Health’s GRABDROP guidelines.

We included women aged 15–49 years (*i.e.* in their reproductive years) with singleton pregnancies, and excluded those who were continuing their pregnancy after leaving hospital; duplicate records; those with missing age; cases of miscarriage; pregnancies at less than 28 weeks’ gestation. The ethics committee of School of Public Health, Jilin University (2024-08-10) approved this study.

### Variables

We considered demographic characteristics (age, marital status, and education) and reported experiences of pregnancies (number of pregnancies, delivery history, caesarean section history, and delivery season that might be associated with method of delivery).

Pregnancy complications included hypertension disorder (defined as systolic blood pressure ≥140 mm Hg and/or diastolic blood pressure ≥90 mm Hg), including pre-existing hypertension, gestational hypertension, preeclampsia, and unclassified hypertension; diabetes (including type 1 and type 2 diabetes, and gestational diabetes); anaemia (haemoglobin concentration of <110 g/L); heart disease; liver disease; kidney disease; hypothyroidism; and hyperthyroidism. Pregnancy complications were diagnosed by clinical doctors identified using ICD-10 codes. Method of delivery encompassed spontaneous labour and caesarean section.

### Statistical analysis

We descriptively summarised the participants’ characteristics and pregnancy complications and used the Rao-Scott χ^2^ test to compare the spontaneous labor and caesarean section groups. We imputed missing values using the K-Nearest Neighbor algorithm.

In the context of pregnancy complications, LCA could help extend the usual binary categorisation of pregnant women as ‘diseased’ and ‘not diseased’ and expand analyses of complications beyond a single condition. Here, we utilised LCA to cluster the pregnancy complications, establishing models by setting the global maximum of the log-likelihood function with a maximum of 1000 iterations. The number of assumed latent classes ranged from two to 10, with five fitted models. We also considered the Akaike information criterion, Bayesian information criterion, and χ^2^ goodness of fit when evaluating model fit. Finally, each class is labelled with an understandable name to enable easier interpretation.

We used logistic regression to examine the associations between pregnancy complications clustered by LCA and method of delivery, fitting three models. Model 1 was a univariate and examined the association between pregnancy complications and caesarean section. Model 2 was multivariate and included significant demographic characteristics for pregnant women as covariates, while model 3 further incorporated important pregnancy complications. A multicollinearity test on independent variables included in the models showed no multicollinearity (variance inflation factor <10).

We used SPSS, version 24.0 (Armonk, New York, USA) and R, version 4.2.2 (R Foundation for Statistical Computing, Vienna, Austria) software with ‘poLCA’ [16], ‘ggplot2’ [17], and ‘DMwR2 [18] packages. A two-sided *P*-value <0.05 indicated significance.

## RESULTS

### Participants’ demographic characteristics

**Figure 1:**
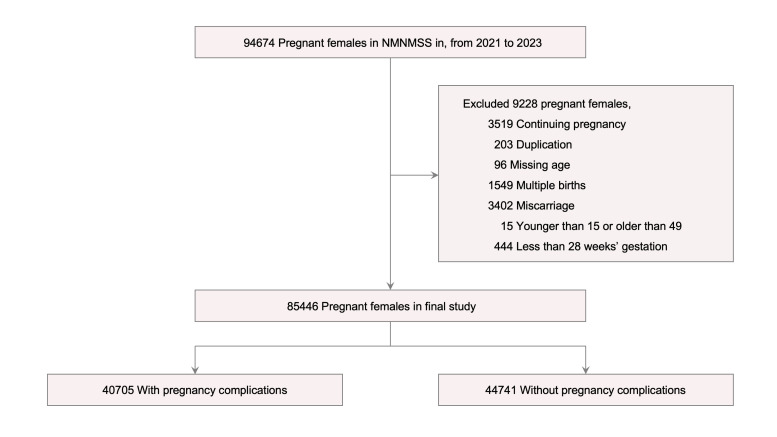
Flowchart of participants selection.

We retrieved data on 94674 pregnant women and included 85 446 in our analysis (**Figure 1**). Of these, 53 916 (63.1%) underwent caesarean section to delivery, 71 122 (83.2%) were aged 20–34 years, 78 540 (91.9%) were married, and 48207 (56.4%) had a college education and above. Only 41 881(49%) were pregnant for the first time, 26 401 (30.9%) had previous deliveries, and 12 397 (14.5%) had undergone a caesarean section. The number of pregnant women delivered varied in different seasons, with 24399 (28.6%) in spring and 18264 (21.4%) in autumn. The spontaneous labour and caesarean section groups differed in age (*P* < 0.001), marital status (*P* < 0.001), education (*P* < 0.001), number of pregnancies (*P* < 0.001), delivery history (*P* < 0.001), caesarean section history (*P* < 0.001), and delivery season (*P* < 0.001) (**Table 1**). There were no significant differences in basic characteristics and pregnancy complications between all and included pregnant women with caesarean section and between pregnant women with and without missing values (Tables S1 and S2 in the **Online Supplementary Document**).

**Table 1:** Descriptive statistics of participants

	Overall (n = 85 446)	Spontaneous labour (n = 31 530)	Caesarean section (n = 53 916)	*P*-value*
**Age in years**				<0.001
15–19	514 (0.6)	307 (59.7)	207 (40.3)	
20–34	71 122 (83.2)	27 965 (39.3)	43 157 (60.7)	
35–49	13 810 (16.2)	3258 (23.6)	10 552 (76.4)	
**Marital status**				<0.001
Not married	6906 (8.1)	1079 (15.6)	5827 (84.4)	
Married	78 540 (91.9)	30 451 (38.8)	480 89 (61.2)	
**Education**				<0.001
Primary school and below	777 (0.9)	269 (34.6)	508 (65.4)	
Junior middle school	11 176 (13.1)	4007 (35.9)	7169 (64.1)	
Senior middle school	25 286 (29.6)	10 018 (39.6)	15 268 (60.4)	
College degree and above	48 207 (56.4)	17 236 (35.8)	30 971 (64.2)	
**Number of pregnancies**				<0.001
1	41 881 (49.0)	15 661 (37.4)	26 220 (62.6)	
2	23 325 (27.3)	9084 (38.9)	14 241 (61.1)	
3	12 174 (14.2)	4295 (35.3)	7879 (64.7)	
≥4	8066 (9.4)	2490 (30.9)	5576 (69.1)	
**Delivery history**				<0.001
No	59 045 (69.1)	21 448 (36.3)	37 597 (63.7)	
Yes	26 401 (30.9)	10 082 (38.2)	16 319 (61.8)	
**Caesarean section history**				<0.001
No	73 049 (85.5)	31 058 (42.5)	41 991 (57.5)	
Yes	12 397 (14.5)	472 (3.8)	11 925 (96.2)	
**Delivery season**				<0.001
Spring	24 399 (28.6)	9097 (37.3)	15 302 (62.7)	
Summer	22 257 (26.0)	8228 (37.0)	14 029 (63.0)	
Autumn	18 264 (21.4)	6497 (35.6)	11 767 (64.4)	
Winter	20 526 (24.0)	7708 (37.6)	12 818 (62.4)	
**Pregnancy with hypertension**				<0.001
No	80 583 (94.3)	30 803 (38.2)	49 780 (61.8)	
Yes	4863 (5.7)	727 (14.9)	4136 (85.1)	
**Pregnancy with diabetes**				<0.001
No	67 653 (79.2)	25 758 (38.1)	41 895 (61.9)	
Yes	17 793 (20.8)	5772 (32.4)	12 021 (67.6)	
**Pregnancy with anaemia**				<0.001
No	63 914 (74.8)	22 548 (35.3)	41 366 (64.7)	
Yes	21 532 (25.2)	8982 (41.7)	12 550 (58.3)	
**Pregnancy with heart disease**				<0.001
No	85 304 (99.8)	31 512 (36.9)	53 792 (63.1)	
Yes	142 (0.2)	18 (12.7)	124 (87.3)	
**Pregnancy with liver disease**				0.623
No	84 892 (99.4)	31 320 (36.9)	53 572 (63.1)	
Yes	554 (0.6)	210 (37.9)	344 (62.1)	
**Pregnancy with kidney disease**				0.024
No	85 402 (99.9)	31 521 (36.9)	53 881 (63.1)	
Yes	44 (0.1)	9 (20.5)	35 (79.5)	
**Pregnancy with hypothyroidism**				<0.001
No	79 559 (93.1)	29 639 (37.3)	49 920 (62.7)	
Yes	5887 (6.9)	1891 (32.1)	3996 (67.9)	
**Pregnancy with hyperthyroidism**				<0.001
No	85 205 (99.7)	31 472 (36.9)	53 733 (63.1)	
Yes	241 (0.3)	58 (24.1)	183 (75.9)	

*Rao-Scott χ^2^ test.

### Prevalence of pregnancy complications

A total of 40705 pregnant women had pregnancy complications, including 4863 (5.7%) with hypertension, 17 793 (20.8%) with diabetes, 21532 (25.2%) with anaemia, 142 (0.2%) with heart disease, 554 (0.6%) with liver disease, 44 (0.1%) with kidney disease, 5887 (6.9%) with hypothyroidism, and 241 (0.3%) with hyperthyroidism. There were significant differences of pregnancy complications (except for liver and kidney disease) between spontaneous labour and caesarean section groups (**Table 1**; Table S3 in the **Online Supplementary Document**).

### LCA of pregnant women

**Figure 2:**
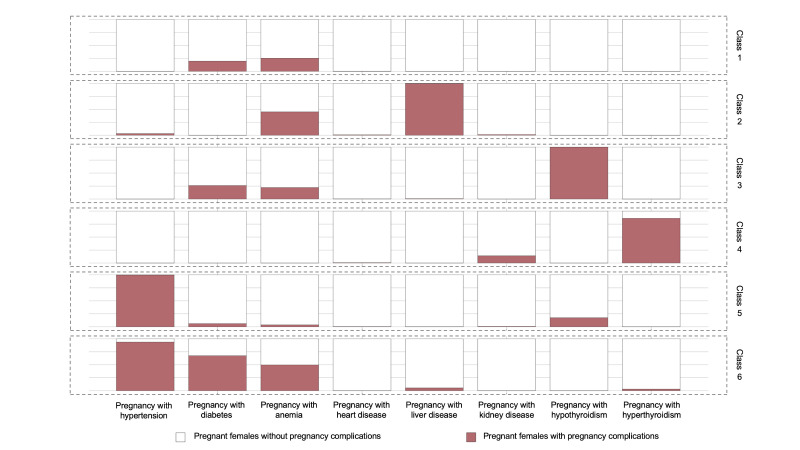
Prevalence of the pregnancy complications in six classes. Class 1: pregnancy with lower incidence of complications. Class 2: pregnancy with anaemia and liver disease. Class 3: pregnancy with diabetes and hypothyroidism. Class 4: pregnancy with hyperthyroidism and kidney disease. Class 5: pregnancy with hypertension and hypothyroidism. Class 6: pregnancy with hypertension, diabetes, and anaemia

We clustered pregnancy complication symptoms into six classes using LCA, labelling them based on prevalence (Table S4 in the **Online Supplementary Document**) as class 1 (pregnancy with lower incidence of complications, meaning below the average in terms of prevalence), class 2 (pregnancy with anaemia and liver disease), class 3 (pregnancy with diabetes and hypothyroidism), class 4 (pregnancy with hyperthyroidism and kidney disease), class 5 (pregnancy with hypertension and hypothyroidism), and class 6 (pregnancy with hypertension, diabetes and anaemia). The highest incidence of caesarean section was in class 5, and the incidence of caesarean section was over 80% in both classes 5 and 6 (**Figure 2**; Figure S1 and Table S5 in the **Online Supplementary Document**).

### Results of logistic regression analysis

We used pregnancies with a lower incidence of complications as the reference for all classes in the logistic regression analyses. In all models, compared with pregnancy with lower incidence of complications, all classes except pregnancy with anaemia and liver disease were significantly associated with caesarean sections. In model 1, pregnancy with diabetes and hypothyroidism (OR = 1.241; 95% CI = 1.171–1.315), pregnancy with hyperthyroidism and kidney disease (OR = 2.208; 95% CI = 1.516–3.215), pregnancy with hypertension and hypothyroidism (OR = 3.581; 95% CI = 3.210–3.994), and pregnancy with hypertension, diabetes, and anaemia (OR = 3.197; 95% CI = 2.869–3.563) were significantly associated with caesarean sections. In model 2, pregnancy with diabetes and hypothyroidism (OR = 1.185; 95% CI = 1.112–1.262), pregnancy with hyperthyroidism and kidney disease (OR = 2.177; 95% CI = 1.456–3.255), pregnancy with hypertension and hypothyroidism (OR = 3.742; 95% CI = 3.334–4.199), and pregnancy with hypertension, diabetes and anaemia (OR=3.335; 95% CI=2.947–3.738) were significantly associated with caesarean sections.

**Figure 3:**
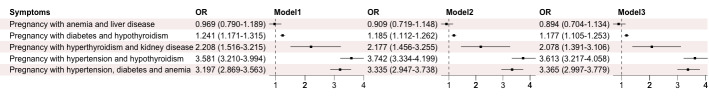
Associations between pregnancy complications and caesarean section. Model 1 was a univariable logistic regression analysis. Model 2 was a multivariable logistic regression analysis adjusting for all significant demographic characteristics, while model 3 further added pregnancy complications significant in model 2. Reference: pregnancy with lower incidence of complications. OR – odds ratio.

In model 2, pregnancy with diabetes and hypothyroidism (OR = 1.198; 95% CI = 1.130–1.271), pregnancy with hyperthyroidism and kidney disease (OR = 2.193; 95% CI = 1.502–3.203), pregnancy with hypertension and hypothyroidism (OR = 3.506; 95% CI = 3.140–3.914), and pregnancy with hypertension, diabetes, and anaemia (OR = 3.082; 95% CI = 2.763–3.438) were significantly associated with caesarean sections. In model 3, caesarean sections were associated with pregnancy with diabetes and hypothyroidism (OR = 1.177; 95% CI = 1.105–1.253), pregnancy with hyperthyroidism and kidney disease (OR = 2.078; 95% CI = 1.391–3.106), pregnancy with hypertension and hypothyroidism (OR = 3.613; 95% CI = 3.217–4.058), pregnancy with hypertension, diabetes, and anaemia (OR = 3.365; 95% CI = 2.997–3.779), but not with pregnancy with anaemia and liver disease (OR = 0.894; 95% CI = 0.704–1.134) (**Figure 3**). = =

## DISCUSSION

We observed a high incidence of caesarean sections in a population of pregnant women from China, with some variations in terms of demographic characteristics and experience of pregnancy. We also found the associations between various pregnancy complications and caesarean sections. Among the pregnancy complication classes, pregnant women with hypertension and hypothyroidism; with hypertension, diabetes, and anaemia; with hyperthyroidism and kidney disease; and with diabetes and hypothyroidism had a higher risk of caesarean section compared to those with a lower incidence of complications. Previous studies have predominantly concentrated on the influence of single pregnancy complications on adverse pregnancy outcomes [3,5]. To address this, we analysed the association between pregnant women with multiple pregnancy complications and caesarean sections.

The rate of caesarean sections in pregnant women was 63.1% in our study, which is higher than reported in countries or regions such as Brazil (55.7%) and Germany (30.5%) [9]. Previous research showed that caesarean section use was markedly high in China among low obstetric risk births [14]. Women’s family and community environment, for example, were found to be associated with factors such as the decision for caesarean section and the outcomes of postpartum care [19]. Fear of labour pain and previous psychological trauma were also found to be significant factors associated with pregnant women’s decision to undergo a caesarean section, with those who used this delivery method tending to believe it was safer [20]. In some cases, women selected the optimal time for the birth of their babies [19]. Our findings indicate a correlation between delivery season and the incidence of caesarean sections. This may be attributable to the varying climatic conditions across different seasons, thereby underscoring the environmental impact on the selection of delivery methods [21]. Previous studies have also found delivery seasons to be associated with the incidence of postpartum depression [22].

Our results also show pregnant women to be susceptible to hypertension, diabetes, and other pregnancy complications, with the combined complications being associated with elevated risks of caesarean section. This phenomenon may be ascribed to the inclination among obstetricians to perform the caesarean section surgery on pregnant women with pregnancy complications [23]. Consequently, we recommended that greater emphasis be placed on the utilisation of caesarean section in pregnant women with pregnancy complications and argue for a reduction in the use of caesarean section without indications in general.

In prior studies, pregnant women with anaemia primarily had iron deficiency anaemia [24]. Hepcidin, a peptide hormone predominantly produced by the liver and is key in regulating iron absorption and homeostasis, controls the balance of homeostatic systems [24]. We observed no significant association between the incidence of caesarean section and pregnancy with anaemia and liver disease. The reason may be that severe anaemia increases perioperative risks, including postpartum haemorrhage, transfusion, and infection, which may theoretically deter clinicians from recommending surgical delivery [25]. Furthermore, anaemia is independently closely related to other adverse pregnancy outcomes, such as an increased risk of placental abruption, maternal shock, and even intensive care unit admission and maternal death [26], highlighting the clinical complexity of managing this high-risk population.

Pregnant women with hypothyroidism and diabetes in our sample had an increased risk of undergoing caesarean sections, which is consistent with previous research [27]. A previous study observed a close association between thyroid dysfunction and diabetes. The prevalence of diabetes in patients with hypothyroidism is higher than that in the general population, and *vice versa* [28]. Requirements for thyroid hormones and daily iodine intake increase during pregnancy, leading to a higher risk of thyroid hormone deficiency [29]. Thyroid diseases are prevalent among pregnant women, with significant implications for maternal and infant health [30]. Thyroid hormones affect early growth and development of the foetus; abnormal thyroid function, for example, is closely associated with adverse developmental outcomes in the foetus [30]. Thyroid dysfunction is associated with various adverse pregnancy outcomes [31]. All other forms of overt hyperthyroidism in pregnancy, except for gestational transient thyrotoxicosis, must be treated to reduce the risks of adverse outcomes, including preeclampsia, low birth weight, miscarriage, and preterm delivery [32]. Furthermore, pregnancy with diabetes has consistently been shown to heighten the probability of caesarean section [5]. Research has also found gestational diabetes mellitus to be associated with an increased risk of preterm delivery, low one-minute Apgar score, macrosomia, and infant born large for gestational age [33]. Consequently, international guidelines advocate preconception counselling for women with pre-existing diabetes mellitus (type 1 or type 2) or obesity [34]. We recommended that they consult with medical experts or nutritionists to develop appropriate nutrition plans and reduce weight in a reasonable manner, and that women with gestational diabetes pay attention to glycaemic control and choose appropriate medication regimens.

A meta-analysis has shown that pregnant women suffering from chronic kidney disease are more likely to undergo caesarean sections [35]. We similarly noted that pregnant women with hyperthyroidism and kidney disease had a higher risk of undergoing caesarean sections. Clinical hyperthyroidism affects between 0.1% and 0.4% of pregnancies [36]; it affects kidney function directly and indirectly through systemic hemodynamic, metabolic, and cardiovascular effects, leading to kidney injury in severe cases [37]. Additionally, kidney diseases, including glomerular diseases, tubulointerstitial disease, and chronic kidney disease, might also be causative factors in hyperthyroidism [38]. Previous studies have demonstrated that the incidence of caesarean section was higher in pregnant women with hyperthyroidism and those with kidney disease [23,39]. The beta subunit of human chorionic gonadotropin, which rises rapidly in early pregnancy, shares structural homology with the thyroid stimulating hormone. This molecular similarity enables human chorionic gonadotropin to exert weak thyrotropic activity at the thyroid stimulating hormone receptor, potentially contributing to gestational thyroid dysfunction [36]. The high incidence of caesarean section in pregnancy with chronic kidney disease might be attributable to the high rate of maternal indications for induction [40]. The presence of chronic kidney disease, in conjunction with hypertension or diabetes, has been demonstrated to increase the probability of adverse pregnancy outcomes, including caesarean sections [41].

Our findings suggest that pregnant women experiencing pregnancy with hypertension and hypothyroidism have a higher probability of undergoing caesarean section. Pregnancy with hypertension was seen to be associated with the occurrence of caesarean sections in previous studies [42] and has been identified as a contributing factor to several adverse pregnancy outcomes such as congenital malformations, intrauterine growth retardation, and even perinatal and foetal death [43]. The incidence of hypertension during pregnancy, including cases complicated by chronic hypertension, is relatively high (5–10%) [44]. In our study, the incidence of pregnancy with hypertension was 5.7%, while the incidence of pregnancy with hypothyroidism was 6.9%.

Pregnancies with hypertension, diabetes, and anaemia, as the three most common complications, were related to one another. Pregnant women with anaemia were more likely to be diagnosed with hypertension and diabetes [45]. Diabetes has previously been identified as a risk factor for hypertension [46]. For noncommunicable diseases such as hypertension, diabetes, and anaemia, a set of assessment measures can be carried out before pregnancy and appropriate preconceptional care can be taken to reduce the incidence of pregnancy-related diseases in women [47]. In addition, pregnancy is now regarded as a physiological stress test that may reveal predispositions to future cardiovascular and endocrine disease [48]. It is therefore necessary that sensitive and reliable early screening and diagnostic tools be developed, and that early treatment be carried out in order to mitigate both immediate pregnancy risks and long-term maternal health burdens.

This study has several strengths. We based our analysis on large-scale provincial monitoring data from a three-year period and used LCA, a robust statistical approach, to classify multiple pregnancy complications. However, we note several limitations, as well. First, we only described associations between various pregnancy complications and caesarean section and could not establish inferences, which would necessitate a different study design. Second, the classes of pregnancy complications in this study were limited, while the combination of pregnancy complications is diverse in reality, meaning we were unable to consider all possible combinations of pregnancy complications.

## CONCLUSIONS

In this retrospective cohort study, we used data from 85 446 pregnant women in China collected between 1 January 2021 and 31 December 2023 using a national surveillance system. We noted a high incidence of caesarean section (63.1%) and found that pregnant women with multiple simultaneous pregnancy complications had a higher incidence of caesarean sections compared to those with fewer complications. These findings suggest that implementing comprehensive, multidisciplinary antenatal clinics for women with ≥2 complications, selecting appropriate delivery methods with the objective of improving the utilisation of caesarean sections.

## Additional material


Online Supplementary Document
