# Supplementary material for: Pregnancy complications and caesarean section: a latent class analysis
Source: J Glob Health. 2026 Apr 3;16:04099. doi: 10.7189/jogh.16.04099 (PMC13045929; doi:10.7189/jogh.16.04099)

Supplement to: Jiang T, Yu G, Kou C, Li W, Liu Y, Meng Y, Wan L, Yi L, Bai W. Pregnancy complications and caesarean section: a latent class analysis. J Glob Health. 2026;16:04099.

Table S1. Comparison between all and included pregnant females in cesarean section

Table S2. Comparison between pregnant females with and without missing values in cesarean section

Table S3. Models of the associations between single pregnancy complications and caesarean section

Table S4. Prevalence of pregnancy complications in different classes

Table S5. Basic characteristics in 6 classes

Table S6. Subgroup analyses

Table S7. Outline of JoGH's Guidelines for Reporting Analyses of Big Data Repositories Open to the Public (GRABDROP) items

Figure S1. Incidence rate of methods of delivery in 6 classes

Table S1. Comparison between all and included pregnant females in cesarean section

Variables		All pregnant females	Included pregnant females	*p*
Age	≤19	215/55575	207/53916	0.969
	20-34	44452/55575	43157/53916	
	≥35	10908/55575	10552/53916	
Marital status	Not married	6103/55636	5827/53916	0.395
	Married	49533/55636	48089/53916	
Education	Primary school and below	529/55643	508/53916	0.970
	Junior middle school	7366/55643	7169/53916	
	Senior middle school	15822/55643	15268/53916	
	College degree and above	31926/55643	30971/53916	
Number of pregnancies	1	27066/55629	26220/53916	>0.999
	2	14688/55629	14241/53916	
	3	8127/55629	7879/53916	
	≥4	5748/55629	5576/53916	
Delivery history	No	38845/55628	37597/53916	0.731
	Yes	16783/55628	16319/53916	
Cesarean section history	No	43448/55637	41991/53916	0.407
	Yes	12189/55637	11925/53916	
Delivery season	Spring	15789/55646	15302/53916	0.875
	Summer	14414/55646	14029/53916	
	Autumn	12101/55646	11767/53916	
	Winter	13342/55646	12818/53916	

Pregnancy with hypertension	No	51294/55646	49780/53916	0.360
	Yes	4352/55646	4136/53916	
Pregnancy with diabetes	No	43289/55646	41895/53916	0.728
	Yes	12357/55646	12021/53916	
Pregnancy with anemia	No	42627/55646	41366/53916	0.646
	Yes	13019/55646	12550/53916	
Pregnancy with heart disease	No	55518/55646	53792/53916	>0.999
	Yes	128/55646	124/53916	
Pregnancy with hepatic disease	No	55298/55646	53572/53916	0.821
	Yes	348/55646	344/53916	
Pregnancy with renal disease	No	55608/55646	53881/53916	0.921
	Yes	38/55646	35/53916	
Pregnancy with hypothyroidism	No	51500/55646	49920/53916	0.814
	Yes	4146/55646	3996/53916	
Pregnancy with hyperthyroidism	No	55455/55646	53733/53916	0.955
	Yes	191/55646	183/53916	

Table S2. Comparison between pregnant females with and without missing values in cesarean section

Variables		With Missing value	Without Missing value	*p*
Age	15-19	207/53916	207/53916	>0.999
	20-34	43157/53916	43157/53916	
	35-49	10552/53916	10552/53916	
Marital status	Not married	5827/53907	5827/53916	>0.999
	Married	48080/53907	48089/53916	
Education	Primary school and below	508/53913	508/53916	>0.999
	Junior middle school	7169/53913	7169/53916	
	Senior middle school	15265/53913	15268/53916	
	College degree and above	30971/53913	30971/53916	
Number of pregnancies	1	26217/53899	26220/53916	>0.999
	2	14236/53899	14241/53916	
	3	7870/53899	7879/53916	
	≥4	5576/53899	5576/53916	
Delivery history	No	37585/53903	37597/53916	0.990
	Yes	16318/53903	16319/53916	
Cesarean section history	No	41989/53916	41991/53916	>0.999
	Yes	11925/53916	11925/53916	
Delivery season	Spring	15302/53916	15302/53916	>0.999
	Summer	14029/53916	14029/53916	
	Autumn	11767/53916	11767/53916	
	Winter	12818/53916	12818/53916	

Pregnancy with hypertension	No	49780/53916	49780/53916	>0.999
	Yes	4136/53916	4136/53916	
Pregnancy with diabetes	No	41895/53916	41895/53916	>0.999
	Yes	12021/53916	12021/53916	
Pregnancy with anemia	No	41366/53916	41366/53916	>0.999
	Yes	12550/53916	12550/53916	
Pregnancy with heart disease	No	53792/53916	53792/53916	>0.999
	Yes	124/53916	124/53916	
Pregnancy with hepatic disease	No	53572/53916	53572/53916	>0.999
	Yes	344/53916	344/53916	
Pregnancy with renal disease	No	53881/53916	53881/53916	>0.999
	Yes	35/53916	35/53916	
Pregnancy with hypothyroidism	No	49920/53916	49920/53916	>0.999
	Yes	3996/53916	3996/53916	
Pregnancy with hyperthyroidism	No	53733/53916	53733/53916	>0.999
	Yes	183/53916	183/53916	

Table S3. Models of the associations between single pregnancy complications and caesarean section

Pregnancy complications	*β*	*SE*	*Wald* χ^2	*p*	OR (95%CI)
Pregnancy with hypertension	1.285	0.043	880.097	<0.001	3.614 (3.320-3.934)
Pregnancy with diabetes	0.199	0.020	101.718	<0.001	1.220 (1.174-1.269)
Pregnancy with anemia	-0.306	0.018	288.701	<0.001	0.736 (0.711-0.763)
Pregnancy with heart disease	1.475	0.265	30.893	<0.001	4.370 (2.598-7.350)
Pregnancy with hepatic disease	-0.070	0.100	0.491	0.483	0.932 (0.766-1.135)
Pregnancy with renal disease	0.813	0.420	3.743	0.053	2.256 (0.989-5.142)
Pregnancy with hypothyroidism	0.145	0.032	21.297	<0.001	1.157 (1.087-1.230)
Pregnancy with hyperthyroidism	0.569	0.163	12.215	<0.001	1.767 (1.284-2.432)

Model was a multivariable logistic regression analysis adjusting for all confounders.

Table S4. Prevalence of pregnancy complications in different classes

Pregnancy complications	Class 1: Pregnancy with lower incidence of complications (*N*=74453)		Class 2: Pregnancy with anemia and liver disease (*N*=389)		Class 3: Pregnancy with diabetes and hypothyroidism (*N*=5438)		Class 4: Pregnancy with hyperthyroidism and kidney disease (*N*=158)		Class 5: Pregnancy with hypertension and hypothyroidism (*N*=2579)		Class 6: Pregnancy with hypertension, diabetes and anemia (*N*=2429)	
	n	(%)	*n*	(%)	*n*	(%)	*n*	(%)	*n*	(%)	*n*	(%)
Pregnancy with hypertension	0	0.0	12	3.1	0	0.0	0	0.0	2572	99.7	2279	93.8
Pregnancy with diabetes	14595	19.6	0	0.0	1407	25.9	0	0.0	154	6.0	1637	67.4
Pregnancy with anemia	18848	25.3	175	45.0	1203	22.1	0	0.0	99	3.8	1207	49.7
Pregnancy with heart disease	117	0.2	3	0.8	11	0.2	1	0.6	10	0.4	0	0.0
Pregnancy with liver disease	0	0.0	387	99.5	35	0.6	0	0.0	2	0.1	130	5.4
Pregnancy with kidney disease	0	0.0	5	1.3	0	0.0	22	13.9	17	0.7	0	0.0
Pregnancy with hypothyroidism	0	0.0	0	0.0	5438	100.0	0	0.0	449	17.4	0	0.0
Pregnancy with hyperthyroidism	32	0.0	0	0.0	0	0.0	136	86.1	0	0.0	73	3.0

Table S5. Basic characteristics in 6 classes

Variables		Class 1<br>*n* (%)	Class 2<br>*n* (%)	Class 3<br>*n* (%)	Class 4<br>*n* (%)	Class 5<br>*n* (%)	Class 6<br>*n* (%)
Age	15-19	490 (0.7)	1 (0.3)	5 (0.1)	0 (0)	6 (0.2)	12 (0.5)
	20-34	62393 (83.8)	301 (77.4)	4462 (82.1)	133 (84.2)	2005 (77.7)	1828 (75.3)
	35-49	11570 (15.5)	87 (22.4)	971 (17.9)	25 (15.8)	568 (22)	589 (24.2)
Marital status	Not married	5714 (7.7)	10 (2.6)	699 (12.9)	31 (19.6)	314 (12.2)	138 (5.7)
	Married	68739 (92.3)	379 (97.4)	4739 (87.1)	127 (80.4)	2265 (87.8)	2291 (94.3)
Education	Primary school and below	669 (0.9)	7 (1.8)	23 (0.4)	3 (1.9)	31 (1.2)	44 (1.8)
	Junior middle school	9914 (13.3)	53 (13.6)	339 (6.2)	17 (10.8)	437 (16.9)	416 (17.1)
	Senior middle school	22173 (29.8)	117 (30.1)	1447 (26.6)	29 (18.4)	749 (29)	771 (31.7)
	College degree and above	41697 (56)	212 (54.5)	3629 (66.7)	109 (69)	1362 (52.8)	1198 (49.3)
Number of pregnancies	1	36284 (48.7)	147 (37.8)	2902 (53.4)	80 (50.6)	1298 (50.3)	1170 (48.2)
	2	20428 (27.4)	113 (29)	1460 (26.8)	36 (22.8)	647 (25.1)	641 (26.4)
	3	10684 (14.3)	74 (19)	684 (12.6)	21 (13.3)	362 (14)	349 (14.4)
	≥4	7057 (9.5)	55 (14.1)	392 (7.2)	21 (13.3)	272 (10.5)	269 (11.1)
Delivery history	No	50897 (68.4)	220 (56.6)	4185 (77)	121 (76.6)	1881 (72.9)	1741 (71.7)
	Yes	23556 (31.6)	169 (43.4)	1253 (23)	37 (23.4)	698 (27.1)	688 (28.3)
Cesarean section history	No	63440 (85.2)	303 (77.9)	4880 (89.7)	142 (89.9)	2229 (86.4)	2055 (84.6)
	Yes	11013 (14.8)	86 (22.1)	558 (10.3)	16 (10.1)	350 (13.6)	374 (15.4)
Delivery season	Spring	21293 (28.6)	108 (27.8)	1528 (28.1)	47 (29.7)	734 (28.5)	689 (28.4)
	Summer	19546 (26.3)	92 (23.7)	1411 (25.9)	39 (24.7)	613 (23.8)	556 (22.9)
	Autumn	15812 (21.2)	80 (20.6)	1169 (21.5)	34 (21.5)	612 (23.7)	557 (22.9)
	Winter	17802 (23.9)	109 (28)	1330 (24.5)	38 (24.1)	620 (24)	627 (25.8)

Method of delivery	Spontaneous labor	28730 (38.6)	153 (39.3)	1828 (33.6)	35 (22.2)	385 (14.9)	399 (16.4)
	Cesarean section	45723 (61.4)	236 (60.7)	3610 (66.4)	123 (77.8)	2194 (85.1)	2030 (83.6)

Table S6. Subgroup analyses

Variables		Overall (N=85446)		Spontaneous labor (N=31530)		Caesarean section (N=53916)		*p*
		n	(%)	*n*	(%)	*n*	(%)	
Age (1)	15-19	514	0.6	307	59.7	207	40.3	<0.001
	20-34	71122	83.2	27965	39.3	43157	60.7	
Age (2)	15-19	514	0.6	307	59.7	207	40.3	<0.001
	35-49	13810	16.2	3258	23.6	10552	76.4	
Age (3)	20-34	71122	83.2	27965	39.3	43157	60.7	<0.001
	35-49	13810	16.2	3258	23.6	10552	76.4	
Education (1)	Primary school and below	777	0.9	269	34.6	508	65.4	0.488
	Junior middle school	11176	13.1	4007	35.9	7169	64.1	
Education (2)	Primary school and below	777	0.9	269	34.6	508	65.4	0.005
	Senior middle school	25286	29.6	10018	39.6	15268	60.4	
Education (3)	Primary school and below	777	0.9	269	34.6	508	65.4	0.513
	College degree and above	48207	56.4	17236	35.8	30971	64.2	
Education (4)	Junior middle school	11176	13.1	4007	35.9	7169	64.1	<0.001
	Senior middle school	25286	29.6	10018	39.6	15268	60.4	
Education (5)	Junior middle school	11176	13.1	4007	35.9	7169	64.1	0.843
	College degree and above	48207	56.4	17236	35.8	30971	64.2	
Education (6)	Senior middle school	25286	29.6	10018	39.6	15268	60.4	<0.001
	College degree and above	48207	56.4	17236	35.8	30971	64.2	
Number of pregnancies (1)	1	41881	49.0	15661	37.4	26220	62.6	<0.001
	2	23325	27.3	9084	38.9	14241	61.1	

Number of pregnancies (2)	1	41881	49.0	15661	37.4	26220	62.6	<0.001
	3	12174	14.2	4295	35.3	7879	64.7	
Number of pregnancies (3)	1	41881	49.0	15661	37.4	26220	62.6	<0.001
	≥4	8066	9.4	2490	30.9	5576	69.1	
Number of pregnancies (4)	2	23325	27.3	9084	38.9	14241	61.1	<0.001
	3	12174	14.2	4295	35.3	7879	64.7	
Number of pregnancies (5)	2	23325	27.3	9084	38.9	14241	61.1	<0.001
	≥4	8066	9.4	2490	30.9	5576	69.1	
Number of pregnancies (6)	3	12174	14.2	4295	35.3	7879	64.7	<0.001
	≥4	8066	9.4	2490	30.9	5576	69.1	
Delivery season (1)	Spring	24399	28.6	9097	37.3	15302	62.7	0.480
	Summer	22257	26.0	8228	37.0	14029	63.0	
Delivery season (2)	Spring	24399	28.6	9097	37.3	15302	62.7	<0.001
	Autumn	18264	21.4	6497	35.6	11767	64.4	
Delivery season (3)	Spring	24399	28.6	9097	37.3	15302	62.7	0.559
	Winter	20526	24.0	7708	37.6	12818	62.4	
Delivery season (4)	Summer	22257	26.0	8228	37.0	14029	63.0	0.004
	Autumn	18264	21.4	6497	35.6	11767	64.4	
Delivery season (5)	Summer	22257	26.0	8228	37.0	14029	63.0	0.212
	Winter	20526	24.0	7708	37.6	12818	62.4	
Delivery season (6)	Autumn	18264	21.4	6497	35.6	11767	64.4	<0.001
	Winter	20526	24.0	7708	37.6	12818	62.4	

Table S7. Outline of JoGH's Guidelines for Reporting Analyses of Big Data Repositories Open to the Public (GRABDROP) items

JoGH guideline item	Purpose
1. Please list all papers published by each co-author in previous three years that were based on secondary analysis of a big data repository	We confirm that none of the co-authors have published papers in the previous three years based on secondary analysis of a big data repository.
2. Please explain the key elements of your study design and the use of the available datasets that make your study an original scientific contribution	This study utilized data from the National Maternal Near Miss Surveillance System (NMNMSS) to investigate the association between caesarean sections and diverse pregnancy complications. This is the study to explore the diverse combinations of pregnancy complications on the incidence of caesarean sections. By employing rigorous statistical methods, we established robust longitudinal associations. The findings of the present study offer novel insights into the associations between the combinations of pregnancy complications and the risk of caesarean section. In addition, it provides a foundation for the development of personalised delivery plans and the optimisation of caesarean section utilisation for pregnant women in a clinical context.
3. Please list all publications that addressed similar research questions in the same dataset and indicate where you cited them in your paper	We confirm that no publications from our research group or other authors have addressed similar research questions (i.e., pregnancy complications and their associations with caesarean section) using the same dataset from the National Maternal Near Miss Surveillance System (NMNMSS).
4. Please explain how you addressed multiple testing through an appropriately rigorous statistical threshold and indicate this in the methods section	While we did not apply a multiple testing correction, we used logistic regression to examine the associations between pregnancy complications clustered by LCA and method of delivery by fitting three models. These models consistently supported our main findings. A two-sided P-value <0.05 indicated significance.

5. Please declare to what extent have AI chatbots been used in developing your paper and to which parts of the paper did they contribute	We declare that AI chatbots were not used at any stage in the development of this manuscript.

Figure S1. Incidence rate of methods of delivery in 6 classes

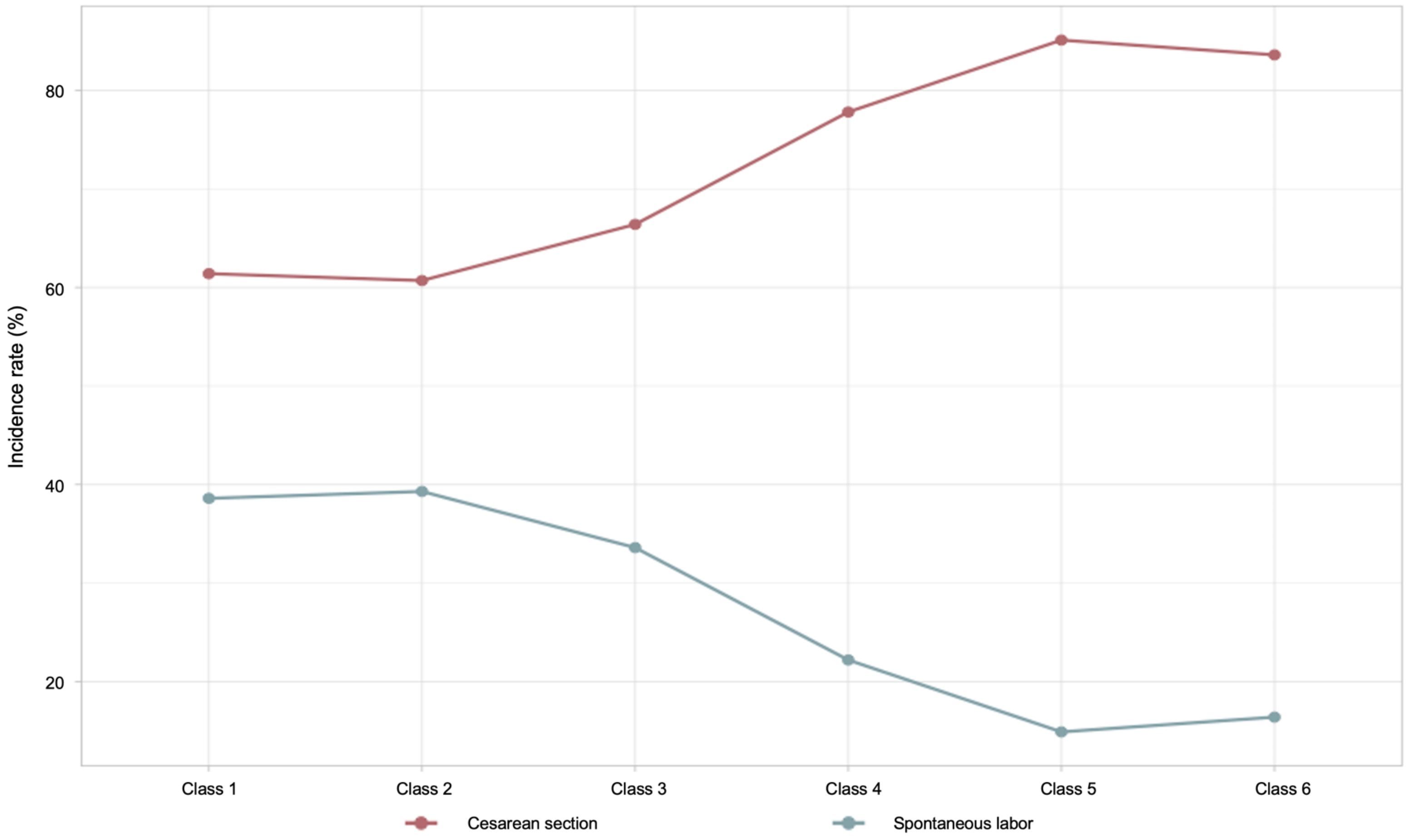

Supplement: Online Supplementary Document [file jogh-16-04099-s001.pdf]
